# The Role of Bile Acid in Improving Glucose Tolerance of Non-Obese Diabetic Rats After Proximal Small Bowel Bypass

**DOI:** 10.3389/fphys.2022.878505

**Published:** 2022-06-16

**Authors:** Zhihua Zheng, Qiang Pang, Xin Luo, Fang Tao, Jinyuan Duan, Jiaqing Cao

**Affiliations:** ^1^ Department of General Surgery, The Second Affiliated Hospital of Nanchang University, Nanchang, China; ^2^ Department of Gastrointestinal Surgery, The Second Affiliated Hospital of Nanchang University, Nanchang, China; ^3^ Department of General Surgery, Gastrointestinal Surgical Institute of Nanchang University, The First Affiliated Hospital of Nanchang University, Nanchang, China

**Keywords:** metabolic surgery, diabetes mellitus, proximal small bowel bypass, bile ileum diversion, bile acid

## Abstract

An increase in bile acid (BA) levels after metabolic surgery is an important mechanism for improving glucose metabolism. However, the mechanisms underlying elevated BA levels and the regulatory mechanism of glucose metabolism remain unclear. In this study, we used the Goto-Kakizaki rat model to investigate the mechanism of BA elevation by comparing side-to-side jejunoileal bypass plus proximal loop ligation (SSJIBL) and bile ileum diversion (BID) as well as to explore the mechanism of BA metabolism in regulating blood glucose. The results showed that the fed blood glucose of rats in both the SSJIBL and BID groups was significantly lower than that of the SHAM group on days 2 and 14 after the operation. The oral glucose tolerance test (OGTT) improved in the SSJIBL and BID groups at day 14 postoperatively. The expression of CYP27A1 in the livers of the SSJIBL and BID groups was significantly increased. In addition, total serum BA levels in the SSJIBL and BID groups were significantly increased. Moreover, serum levels of lithocholic acid (LCA) and deoxycholic acid (DCA) were significantly higher in the SSJIBL group than in the SHAM group and negatively correlated with the area under the glucose tolerance curve (AUC-OGTT). In conclusion, increased BA synthesis may be an important cause of elevated total serum BA levels, and LCA and DCA are closely associated with improved glucose metabolism.

## Introduction

The incidence of diabetes mellitus continues to increase dramatically worldwide (Manne-Goehler et al., 2019) and treatment is no longer limited to drugs alone. Metabolic surgery (MS) is gradually being used to treat diabetes with excellent results (Knop, 2009; Harris et al., 2019; Yan et al., 2019) and is the most effective cure. MS is performed in various ways, including classical jejunoileal bypass (Wang et al., 2016), duodenal-jejunal bypass (Zhang et al., 2016), Roux-en-Y gastric bypass (RYGB) (Noel et al., 2021), and sleeve gastrectomy (Wang et al., 2017). The improvement in blood glucose levels after these procedures was initially thought to be achieved solely through mechanisms such as malnutrition malabsorption and caloric restriction. However, numerous researchers have observed that the improvement of blood glucose after surgery was accompanied by phenomena such as elevated serum bile acid (BA) levels and confirmed that BA plays an important role in improving blood glucose metabolism (Kaska et al., 2016; Chen et al., 2019; Yan et al., 2019).

BA is converted from cholesterol in the liver, whereas the produced BA is stored in the gallbladder which excretes BA into the intestine in response to food stimulation to facilitate the digestion and absorption of lipids and fat-soluble vitamins. In the distal ileum, approximately 95% of BA return to the liver through the portal vein, a process known as enterohepatic circulation. The remaining 5% is eliminated via the feces. A small proportion of BA reabsorbed via the portal vein enter the peripheral circulation as serum bile acids. BA participates in glucose metabolism as signaling molecules mainly through the nuclear hormone farnesoid X receptor (FXR) and Takeda G protein receptor 5 (TGR5). BA regulates glucose homeostasis after Roux-en-Y gastric bypass (RYGB) surgery by promoting insulin secretion through the BA-FXR-glucagon-like peptide (GLP-1) axis (Li et al., 2020), or by activating FXR to inhibit hepatic gluconeogenesis. McGavigan et al. observed that BA after SG could activate the TGR5-GLP-1 pathway, thus improving glucose metabolism (McGavigan et al., 2017).

Notably, MS involves alterations in the physiology and anatomy of the small intestine. Some studies have shown the effects of MS on intestinal physiology, neural signaling, intestinal incretin secretion, BA metabolism, and microbiota changes, which may be closely related to the improvement in glucose metabolism after MS (Breen et al., 2012; Affinati et al., 2019). An increasing number of researchers believe that the small intestine plays a key role in regulating glucose metabolism (Duca et al., 2021). Previously, our team designed a side-to-side jejunoileal bypass plus proximal loop ligation (SSJIBL), which bypasses the proximal small intestine and can significantly improve glucose metabolism with elevated total serum BA (TBA) levels (Duan et al., 2015). However, the mechanism of elevated BA levels and the regulation of glucose metabolism are unclear. Therefore, in the current study, we used the Goto-Kakizaki (GK) rat model and divided it into two surgical groups, SSJIBL and bile ileum diversion (BID), to investigate the mechanism of elevated BA and the role of BA metabolism in glucose regulation.

## Materials and Methods

### Animals

All animal experiments were conducted in accordance with the Animal Experiment Guide of Nanchang University and approved by the Animal Ethics Committee of Nanchang University. The current study was conducted on 30 male GK rats, aged 10–12 weeks upon arrival. All animals were provided by Slac Laboratory Animal Co., Ltd. (Shanghai, China). Rats with random blood glucose ≥16.7 mmol/L were considered diabetic. Finally, 18 diabetic rats were randomly selected for subsequent experiments.

### Experimental Design

Eighteen GK rats were randomly assigned to three groups: SHAM, SSJIBL, and BID, with eight rats (*n* = 6) in each group. Body weight (BW), food intake (FI), fed blood glucose (FEG), and oral glucose tolerance test (OGTT) were measured in all rats before surgery. Subsequently, the three groups underwent relevant surgical operations. Postoperatively, all the rats had free access to water and food. BW, FI, and FEG were measured daily after surgery. Blood samples and liver tissues were collected on postoperative day 15 for subsequent testing. Finally, all the rats were euthanized.

### Surgical Techniques

All rats were fasted for 12 h before surgery. A total of 10% chloral hydrate (300 m/kg) was used for anesthesia, and the abdominal hair was carefully shaved, followed by routine disinfection. A median abdominal incision (∼4 cm) was then made.

**FIGURE 1:**
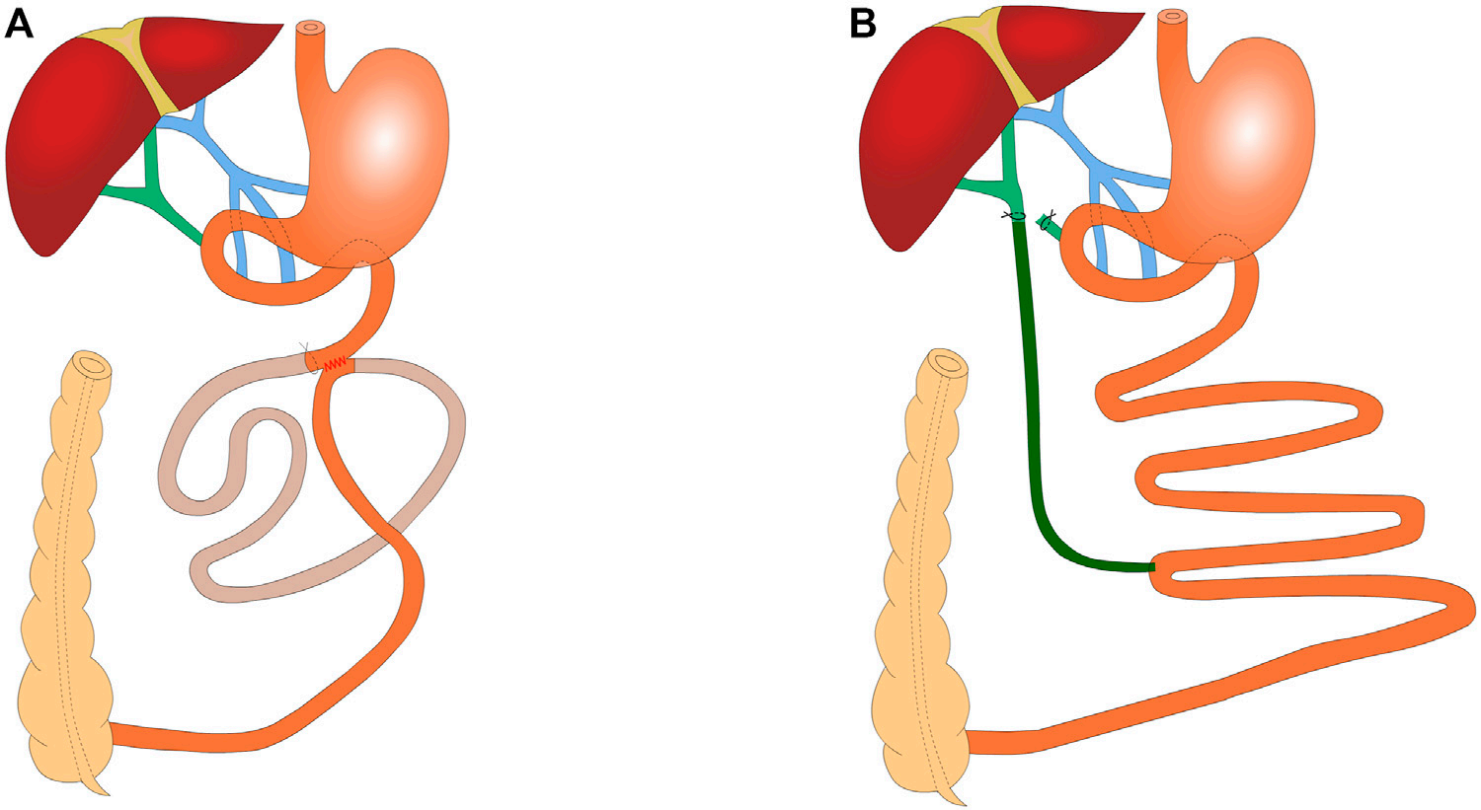
Schematic of SSJIBL (*n* = 6) and BID (*n* = 6) **(A)** SSJIBL procedure to restore intestinal continuity with a lateral anastomosis using the distal 6 cm of the ligament of Treitz and the proximal 30 cm of the ileocecal valve as reference points and a ligation at the proximal end of the bypassed intestine (∼1 cm from the anastomosis), with ∼60% of the entire small intestine being bypassed. **(B)** BID procedure: the lower portion of the common bile duct was ligated first, and the one end of the common bile duct was placed 0.5 cm above the ligation site with a silicone hose; the other end of the silicone hose was placed in the ileum at 30 cm from the ileocecal portion.

SSJIBL: This procedure bypasses approximately 60% of the length of the entire small bowel, with details of the procedure used in a previous study (Duan et al., 2015). Briefly, a point 30 cm proximal to the ileocecal valve and another point 6 cm distal to the ligament of Treitz were used as the reference points. Bowel continuity was restored by side-to-side anastomosis between the proximal jejunum and the ileum. Luminal occlusion was performed in the first portion of the bypassed segment using ligation with 0 silk sutures (Figure 1A).

BID: The abdominal cavity was fully exposed upon its opening. The common bile duct was found along the top of the duodenum. The lower part of the common bile duct was ligated with 7–0 nylon sutures and the common bile duct was ligated again at 0.5 cm above the ligature, without ligature for the time being. Under the microscope, the common bile duct was cut in the middle of the two lines, and the silicone hose (0.5 mm inner diameter/1 mm outer diameter) was carefully placed into the common bile duct (∼0.5 cm). When yellowish clear bile dripped from the drainage tube, the bile duct was trapped and fixed. At 30 cm from the ileocecum, the opposite side of the intestinal canal was incised along the mesenteric margin, and the other end of the silicone hose was placed into the ileum (∼1.5 cm into the ileum), keeping the drainage tube parallel to the ileum for 1 cm to prevent fracture (Figure 1B).

SHAM: After entering the abdominal cavity, the intestine was exposed as in the surgical group, but no manipulation was performed. The operative time was prolonged to induce a degree of anesthetic stress comparable to that experienced by operated rats.

After the relevant surgical procedures were completed, the entire small intestine was returned to the abdominal cavity. After confirming that no bleeding or leakage occurred in the abdominal cavity, the abdominal incision was closed with 3–0 silk sutures. All rats were allowed to drink freely for 2 h after the operation and eat normally 24 h later.

### Body Weight and Food Intake

BW and FI were measured at baseline and daily postoperatively until the end of 14 days.

### Fed Blood Glucose and Oral Glucose Tolerance Test

Blood glucose concentrations were measured using an electronic glucometer (Accu-Chek Performa^®^, Roche Diagnostic, Switzerland) in the blood obtained from the tail vein of awake rats. For the FEG, the blood glucose level was measured at 08:00 before surgery and daily postoperatively.

OGTT was performed before surgery and at 2 and 14 days postoperatively. All the rats were fasted for 12 h and fed intragastrically with 10% glucose (1 g/kg). Blood glucose levels were measured at 0, 15, 30, 60, 90, 120, and 180 min. The area under the glucose tolerance curve (AUC-OGTT) was calculated.

### Sample Collection

Fourteen days after surgery, fecal pellets were collected from all rats within 24 h, labeled, and placed in a −80°C freezer. After all rats were anesthetized, the liver tissue was removed and rinsed thoroughly with sterile saline, cut into small pieces, and placed in −80°C freezing tubes for later analysis. Blood was collected from the abdominal aorta using a coagulation-promoting vacuum blood collection tube and then centrifuged (3,000 rpm for 10 min) at room temperature for 2 h. Then, the supernatant was transferred to a freezing tube and stored at −80°C.

### qPCR

Total RNA was extracted from the liver tissue using TRIzol reagent (Invitrogen, Waltham, MA, United States). cDNA was synthesized using the RT First-Strand cDNA Synthesis Kit (Servicebio, Wuhan, China). the relative level of mRNA amplification was determined using SYBR Green qPCR Master Mix (High ROX) (Servicebio) in accordance with the manufacturer’s instructions. GAPDH Primer Assay was used as a house keeping. The following primers were used: Cyp7a1′5′-GGCATCTCAAGCAAACACC’T-3′ (forward) an’ 5′-GC TGTGCG GATATTCA AGG’T-3′ (reverse); Cyp27a1′5′-CTGCCCTTTTGGAAGCGA’A-3′ (forward) an’ 5′-TTG​GAT​GTC​GTG​TCT​ACC’C-3′ (reverse); Cyp8b1′ 5′-TTCAAGTACAATCGG TTCCT’A-3′ (forward) and 5′-GGT​CCA​CCA​GTT​CAA​AGT​CAA​A-3′ (reverse); BA conjugational enzymes (BACS),5′-GGCTGCTCAATCACAAATAGG-3′(forward) and 5′-CAG​AAA​TGG​ACT​TGG​ACG​G-3′ (reverse); GAPDH, 5′-CTG​GAG​AAA​CCT​GCC​AAG​TAT​G-3′ (forward) and 5′-GGT​GGA​AGA​ATG​GGA​GTT​GCT-3′ (reverse).

### Lipid Homeostasis and Total Serum Total Bile Acids

Serum samples were removed from the -80°C freezer and then centrifuged (3,000 rpm for 10 min) at room temperature for 2 h. The supernatant was used for the assay. Total serum cholesterol (CHOL), triglyceride (TG), high-density lipoprotein (HDL), low-density lipoprotein (LDL), and TBA levels were measured using an automatic biochemical analyzer (Chemray 800). The tests and analyses were conducted in a biochemical laboratory.

### Targeted BA Quantification

BA was quantitatively analyzed using ultra-high-performance liquid chromatography-electrospray ionisation-tandem mass spectrometry (UPLC-MS). The chromatographic column was a Phenomenex Kinetex C18 (2.1 mm × 100 mm, 2.6 µm). The binary gradient elution system consisted of (A) water (containing 0.1% formic acid, v/v) and (B) acetonitrile (containing 0.1% formic acid, v/v). Separation was achieved using the following gradient: 0 (66:34, v/v), 0.5 (66:34, v/v), 9 (49:51, v/v), 13 (5:95, v/v), 14 (5:95, v/v), 14.1 (66:34, v/v), and 15 min A/B (66:34, v/v). The flow rate was 0.4 ml/min, and the column temperature was 45°C. The injection volume was 5 µL. The MS parameters were as follows: ion source temperature, 450°C; negative ion spray voltage, 4500 V; curtain gas, 35 PSI; collision-activated dissociation; and medium. The acquired UPLC-MS raw data were analyzed using Progenesis QI software (Waters Corporation, Milford, MA, United States).

Serum BA: A total of 100 µL serum was added to a 1.5 ml Eppendorf tube with 10 µL 2-chloro-l-phenylalanine (0.3 mg/ml) dissolved in methanol as the internal standard, and the tube was vortexed for 10 s. Subsequently, 400 µL ice-cold mixture of methanol and acetonitrile (2/1, v/v) was added, and the mixtures were vortexed for 1 min and centrifuged at 13,000 rpm at 4°C for 10 min. A total of 400 µL of supernatant in a brown glass vial was dried in a freeze-concentration centrifugal dryer. Next, 200 µL of a mixture of methanol and water (1/4, v/v) was added to each sample. The samples were vortexed for 30 s and incubated at 4°C for 2 min. The samples were centrifuged at 13,000 rpm at 4°C for 10 min. The supernatants (200 µL) from each tube were collected using crystal syringes, filtered through 0.22 µm microfilters, and transferred to LC vials.

Fecal BA: A total of 50 mg of accurately weighed sample was transferred to a 1.5 ml Eppendorf tube. Two small steel balls are added to each tube. Exactly 200 µL of the extraction solvent with methanol/water (1/1, v/v) was added to each sample. Samples were ground at 60 Hz for 2 min and stored at −20°C for 30 min. The extract was centrifuged at 13,000 rpm at 4°C for 10 min. Subsequently, 1 ml of the supernatant was placed in a brown glass vial and dried in a freeze-concentration centrifugal dryer. Subsequently, 200 µL of a mixture of methanol and water (1/1, v/v) was added to each sample. The samples were vortexed for 30 s and centrifuged at 13,000 rpm at 4°C for 5 min. The supernatants (200 µL) from each tube were collected using crystal syringes, filtered through 0.22 µm microfilters, and transferred to LC vials. The vials were stored at −80°C until UPLC-MS analysis.

### Statistics

Data are expressed as mean ± standard error of the mean (SEM). The area under the curve (AUC) was calculated using trapezoidal integration. BW, FI, FEG and OGTT over time were analyzed using two-way ANOVA. AUCs, lipid homeostasis, BA-related enzyme profiles, serum and fecal BAs were compared using one-way ANOVA. Representative BA and glucose homeostasis correlation index were analyzed by Pearson’s correlation analysis. Tukey’s test was performed for pairwise comparisons between groups. All analyses were performed using GraphPad Prism version 9.0.2. *p* < 0.05 represented statistical difference.

## Results

### Body Weight and Food Intake

**FIGURE 2:**
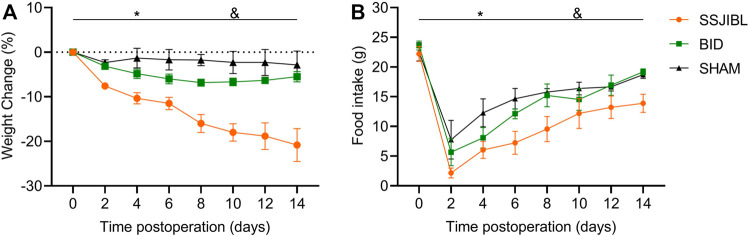
BW **(A)** and FI **(B)** of rats after surgery (*n* = 6 for SSJIBL, *n* = 6 for Sham, and *n* = 6 for BID). Body weight (BW); food intake (FI). *p < 0.05 for SSJIBL versus SHAM group; ^&^ *p* < 0.05 for SSJIBL versus BID.

No statistical difference was observed in BW or FI of the rats in the SHAM, SSJIBL, and BID groups before surgery. Two-way ANOVA showed that the rats in the SSJIBL group exhibited a lower global BW than those in the SHAM and BID groups after surgery (both *p* < 0.05), while there was no significant difference between the SHAM and BID groups (Figure 2A).

The rats in the SSJIBL group exhibited lower FI than those in the BID and SHAM groups (both *p* < 0.05). FI of rats in the BID group decreased to the lowest level on the second day after surgery and then gradually increased to the same level as that in the SHAM group (Figure 2B).

### Fed Blood Glucose

**FIGURE 3:**
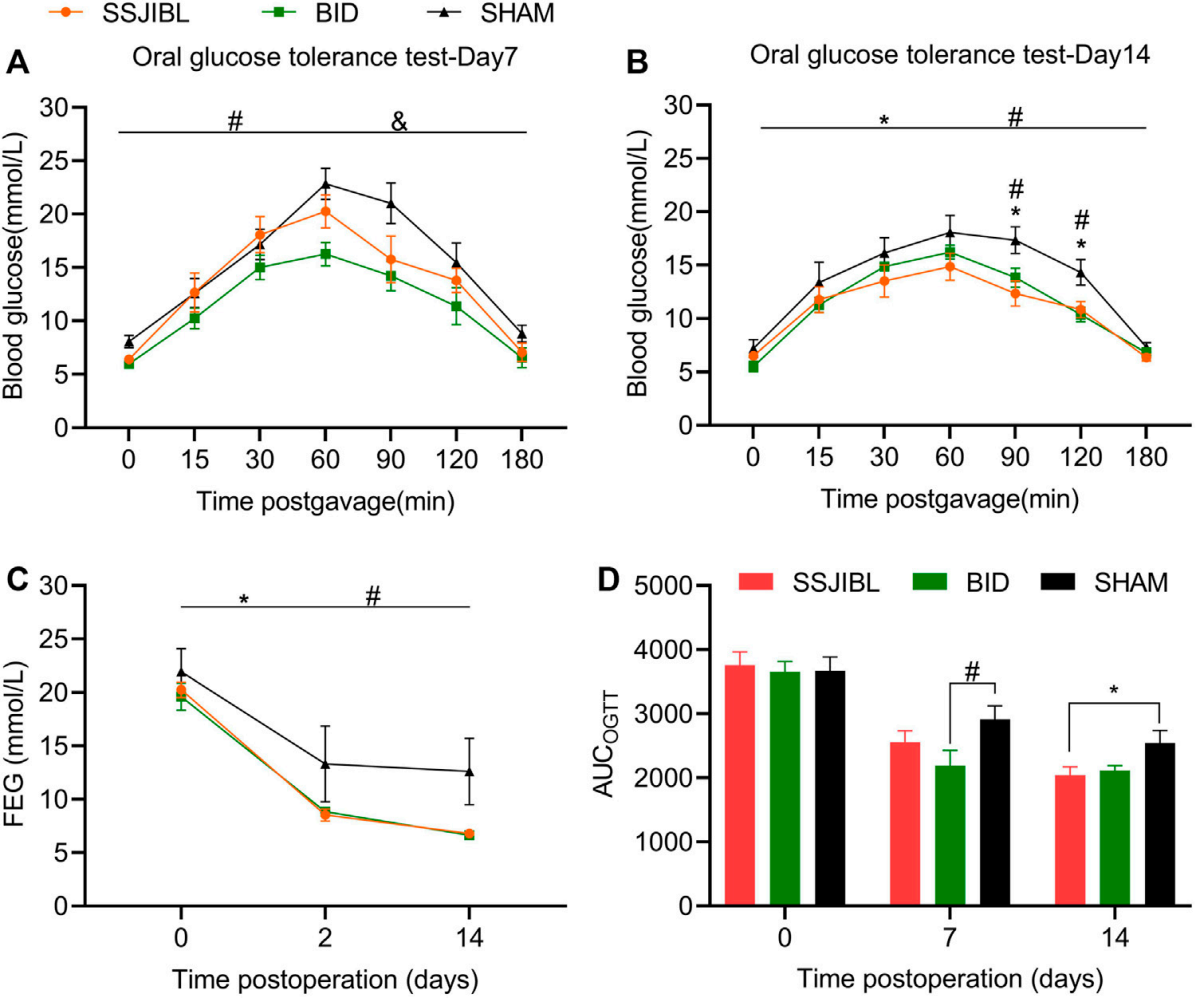
OGTT and FEG. **(A)** OGTT 7 days after surgery (*n* = 6 for SSJIBL, *n* = 6 for Sham, and *n* = 6 for BID). All rats were fasted for 12 h and fed with 10% glucose (1 g/kg) intragastrically. **(B)** OGTT 14 days after surgery. **(C)** Average FEG levels of rats in all groups before, 2 and 14 days after the surgery. **(D)** AUC-OGTT. The area under the curve (AUC) was calculated using trapezoidal integration. Fed blood glucose (FEG); Oral glucose tolerance test (OGTT). ^*^ *p* < 0.05 for SSJIBL versus SHAM group; ^#^ *p* < 0.05 for BID versus SHAM group; ^&^ *p* < 0.05 for SSJIBL versus BID.

No difference was observed in postprandial blood glucose levels among the SHAM, SSJIBL, and BID groups of rats before surgery. At 2 and 14 days post-operation, the FEG decreased significantly in rats in the SSJIBL and BID groups compared with that in the SHAM group (both *p* < 0.05), but no difference was recorded between the SSJIBL and BID groups (Figure 3C).

### Oral Glucose Tolerance Test

No significant difference was observed in OGTT between the preoperative SSJIBL, BID, and SHAM groups. Seven days post-operation, rats in the BID group had significantly improved oral glucose tolerance compared with those in the SHAM and SSJIBL groups (both *p* < 0.05), exhibiting lower blood glucose concentrations (Figure 3A). At 14 days postoperatively, the SSJIBL and BID groups showed significantly improved glucose tolerance compared to the SHAM group, exhibiting lower blood glucose concentrations at 9 and 120 min (Figure 3B). In addition, the AUC-OGTT of the SSJIBL group was significantly improved compared to that of the SHAM group 14 days after surgery (Figure 3D).

### Expression of BA-Related Enzyme Profiles

**FIGURE 4:**
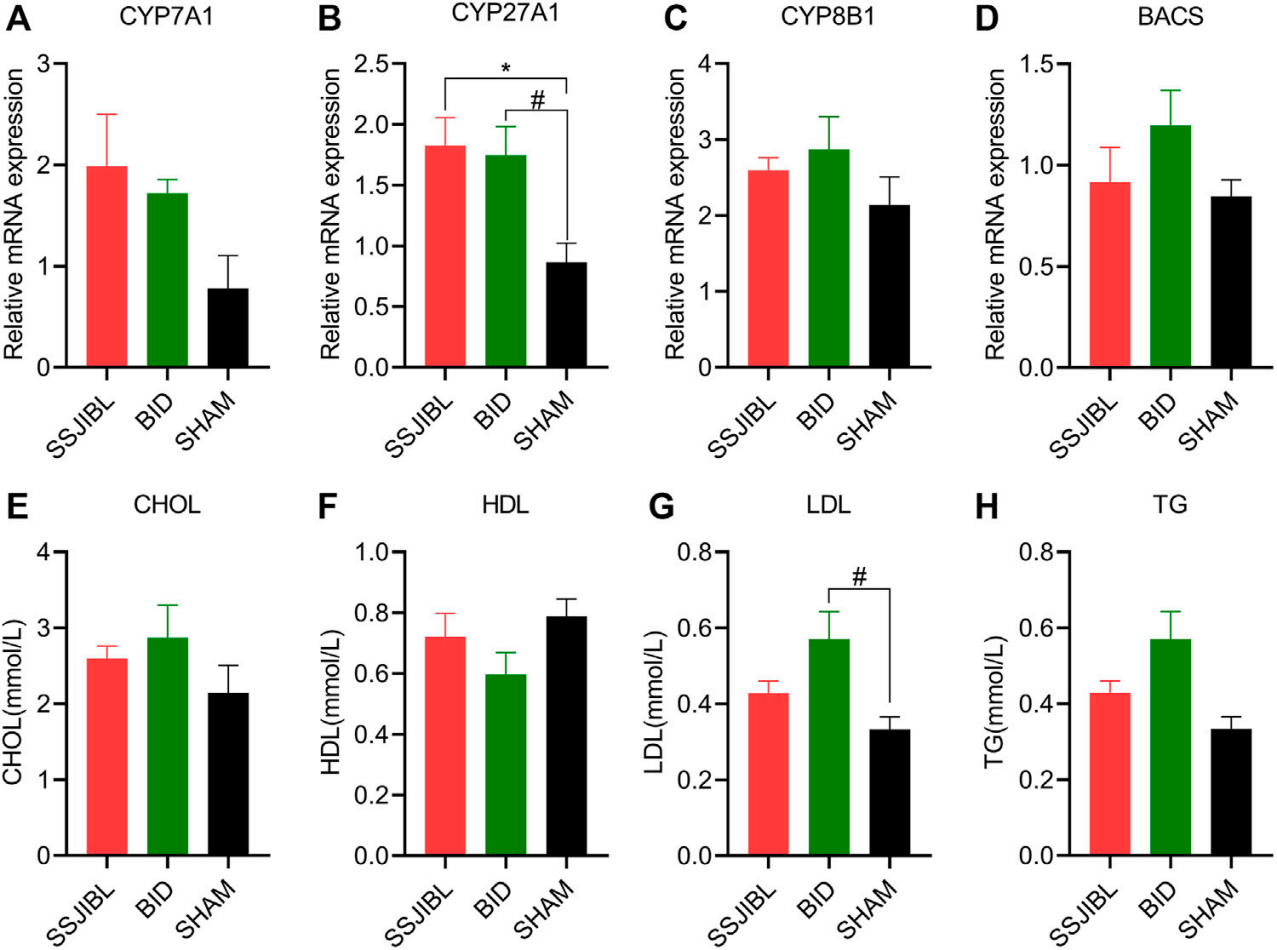
**(A–D)** Relative mRNA expression of enzymes CYP7A1, CYP27A1, CYP8B1, and BACS in the liver (*n* = 6 for SSJIBL, *n* = 6 for Sham, and *n* = 6 for BID). GAPDH was used as an internal control. **(E–H)** Total TG, CHOL, HDL, and LDL levels in blood. TG, triglyceride; CHOL, cholesterol; HDL, high-density lipoprotein; LDL, low-density lipoprotein. ^#^ *p* < 0.05 for BID versus SHAM group. ^*^ *p* < 0.05 for SSJIBL versus SHAM group.

To better understand the expression of hepatic BA synthases (CYP7A1, CYP27A1, and CYP8B1) and binding enzymes (BACS), qPCR was performed on the liver tissues. The expression of CYP27A1 was significantly increased in the SSJIBL and BID groups compared to that in the SHAM group (both *p* < 0.05) (Figure 4B). In addition, although there was a trend of elevated expression of CYP7A1, CYP8B1, and BACS in the SSJIBL and BID groups, these differences were not statistically significant (Figures 4A,C,D).

### Lipid Homeostasis

The results of the automatic biochemical analyzer showed that TG, CHOL, and HDL in the SSJIBL and BID groups had no statistical significance compared with the SHAM group, and only LDL in the BID group was significantly higher than that in the SHAM group (*p* < 0.05) (Figures 4E–H).

### Serum and Fecal BA Profiles

**FIGURE 5:**
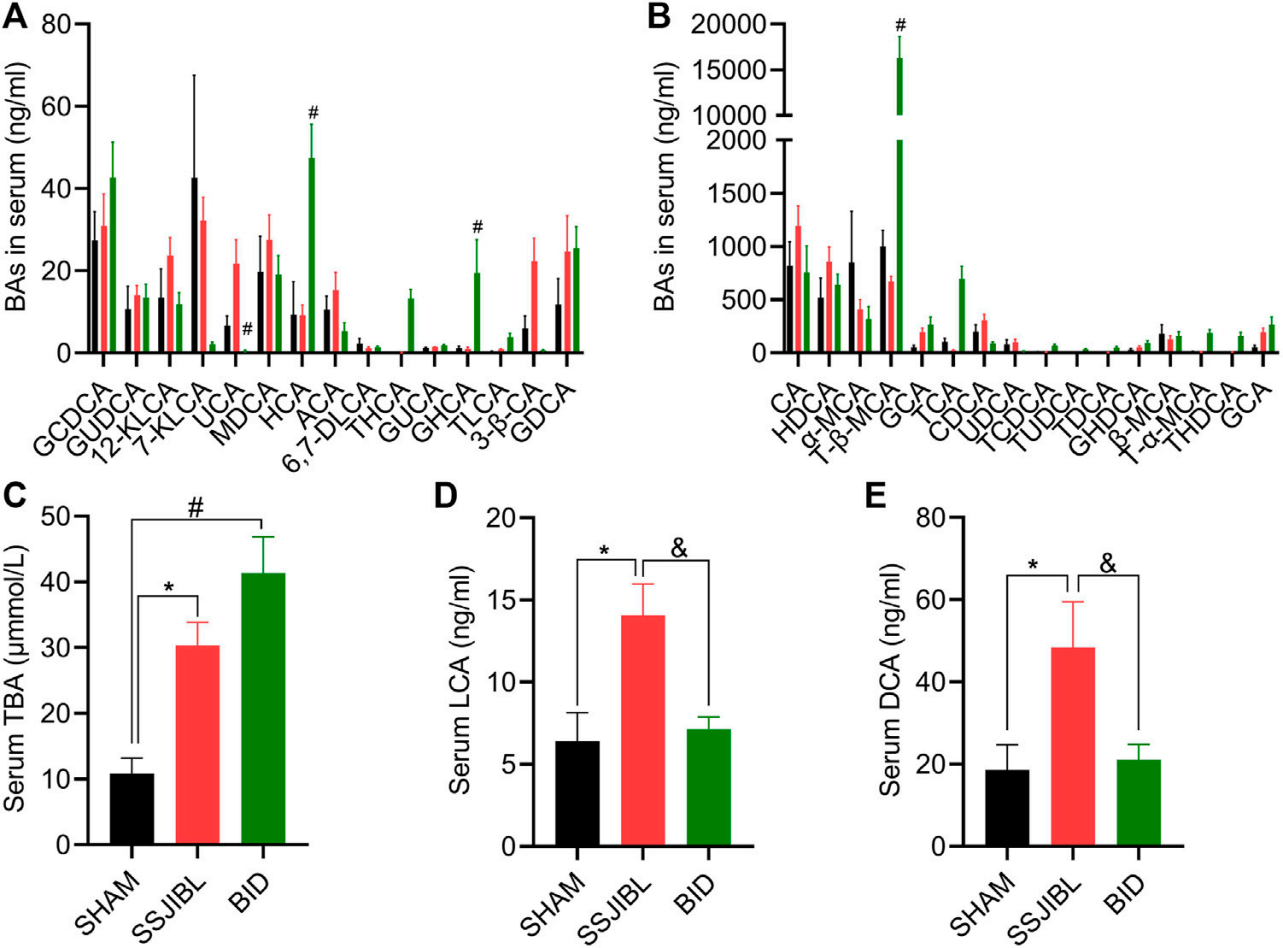
Serum BA composition. **(A,B)** Absolute values of each BA component in serum at days 14 after surgery. **(C)** Total serum total bile acids (TBA). **(D,E)** Absolute values of LCA and DCA. LCA, lithocholic acid; GLCA, glycolithocholic acid; TLCA, taurolithocholic acid; TDCA, taurodeoxycholic acid; KLCA, 12-ketolithocholic acid; 6,7-DLCA, 6,7-diketolithocholic acid; GUCA, glycoursocholanic acid; 3β-UDCA, 3beta-ursodeoxycholic acid; 7-KLCA, 7-ketolithocholic acid; 3β-CA, 3beta-cholic acid; USCA, ursocholic acid; ACA, allocholic acid; nor-DCA, nor-desoxycholic acid; CDCA, chenodeoxycholic acid; DCA, deoxycholic acid; UDCA, ursodeoxy-cholic acid; GDCA, glycodeoxycholic acid; GCDCA, glycochenodeoxycholic acid; TUDCA, tauroursodeoxycholic acid; TCDCA, taurochenodesoxycholic acid; GHDCA, glycohyodeoxycholic acid; β-MCA, beta-muricholic acid; GHCA, glycohy-ocholic acid; MDCA, murideoxycholic acid; HCA, hyocholic acid; CA, cholic acid; GUDCA, glycoursodeoxycholic acid; GCA, glycocholic acid; TCA, taurocholic acid; 23-NCA, 23-norcholic acid; HDCA, hyodeoxycholic acid; α-MCA, alpha-muricholic acid; THCA, taurohyocholic acid; T-α-MCA, tauro-alpha-muricholic acid; THDCA, taurohyodeoxycholic acid; T-β-MCA, tauro-beta-muricholic acid. #*p* < 0.05 for BID versus SHAM group. *p < 0.05 for SSJIBL versus SHAM group; ^&^ *p* < 0.05 for SSJIBL versus BID group.

Serum BA: TBA levels were significantly higher in the SSJIBL and BID groups than in the SHAM group (Figure 5C). Compared with the SHAM and BID groups, serum lithocholic acid (LCA) and deoxycholic acid (DCA) levels were significantly elevated in the SSJIBL group rats (all *p* < 0.05) (Figures 5D,E).

**FIGURE 6:**
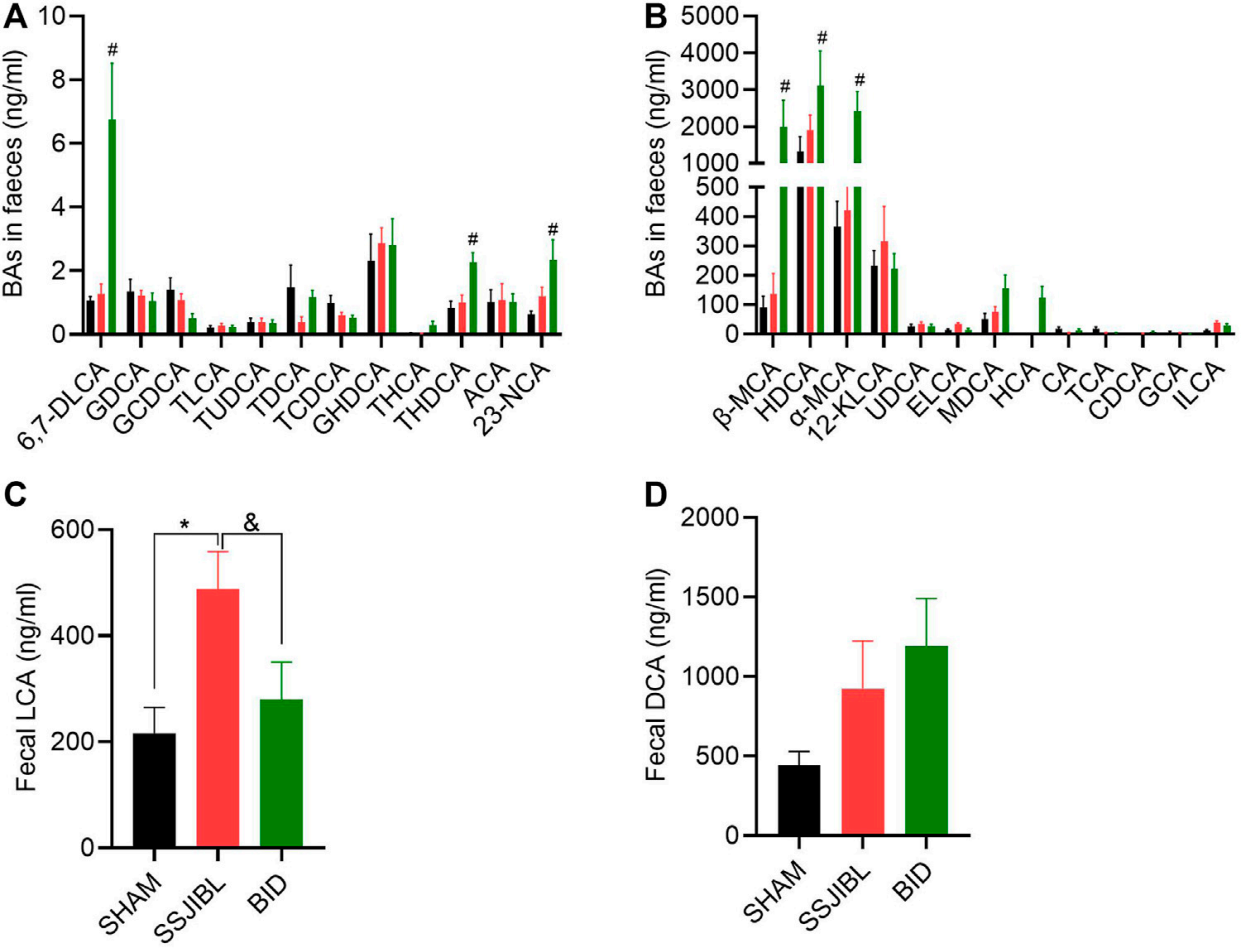
Fecal BA composition. **(A,B)** Absolute values of each BA component in feces at days 14 after surgery. **(C,D)** Absolute values of LCA and DCA. ELCA, Epiallolithocholic Acid; ILCA, Isolithocholic Acid. #*p* < 0.05 for BID versus SHAM group; *p < 0.05 for SSJIBL versus SHAM group; ^&^ *p* < 0.05 for SSJIBL versus BID group.

Fecal BA: The LCA of the SSJIBL group was significantly higher than that of the SHAM and BID groups (Figure 6C). Compared to the SHAM group, there was an increasing trend of DCA in feces in both the SSJIBL and BID groups, but the difference was not statistically significant (Figure 6D).

**FIGURE 7:**
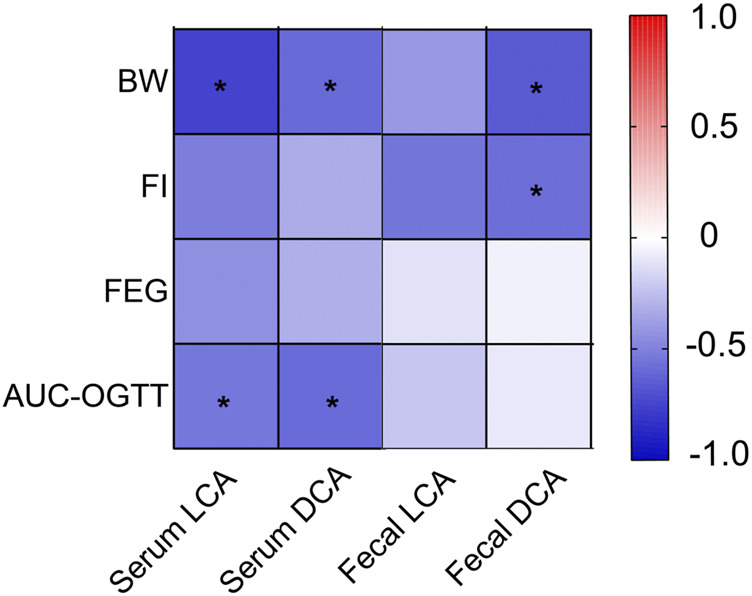
Heatmap analysis of the Pearson correlation of serum and fecal BA and glucose homeostasis-related indexes. Red represents a positive correlation and blue indicates a negative correlation. Body weight (BW); Food intake (FI). *p < 0.05.

We performed a heatmap analysis of Pearson correlations for representative BA and glucose homeostasis-related indices. The results revealed that serum levels of LCA and DCA were negatively correlated with AUC-OGTT (all *p* < 0.05), whereas serum levels of LCA, DCA, and fecal DCA levels were negatively correlated with BW (both *p* < 0.05) (Figure 7).

## Discussion

In the present study, we investigated the mechanism of elevated BA levels and the role of BA metabolism in improving blood glucose by comparing the efficacy of the SSJIBL group with that of the BID group using the GK rat model. We found that the rats in the SSJIBL group had a significant decrease in postoperative BW, whereas the rats in the BID group did not show significant weight loss. The SSJIBL and BID groups showed a significant improvement in FEG levels, an increase in serum TBA levels, and a significant increase in the expression of BA synthase CYP27A1. In addition, we analyzed the correlation between BA composition and glucose homeostasis-related indices and found that serum levels of LCA and DCA were negatively correlated with AUC-OGTT.

Although targeted metabolomics could not determine serum TBA levels, we measured TBA levels using an automatic biochemical analyzer, showing that they were significantly elevated after SSJIBL and BID. In general, BA levels are elevated for several reasons: increased BA synthesis, decreased BA excretion, and increased BA absorption. Ferrannini et al. observed that the mechanism of BA elevation may be different in various bariatric surgeries, with the elevation of BA after biliopancreatic diversion because of increased synthesis and RYGB to a different mechanism (Ferrannini et al., 2015). In the present study, the expression of postoperative BA synthase increased in both surgical groups of rats, with a significant increase in CYP27A1 expression. CYP7A1, CYP27A1, and CYP8B1 are involved in BA synthesis and CYP7A1 and CYP27A1 are key enzymes in the classical and alternative pathways of BA synthesis, respectively. These results suggest that SSJIBL and BID can elevate serum BA levels by increasing BA synthesis. Although we were unable to measure total fecal BA levels in the current study, an increasing trend in BA excretion was observed based on the detection of fecal BA components. Therefore, decreased excretion cannot explain the increase in TBA levels. Given that the expression of BA transport receptors was not detected, the role of BA reabsorption in elevating TBA levels warrants further investigation.

The present study showed a decrease in BW and FI in the SSJIBL group compared with the SHAM group, but not in the BID group. Interestingly, the changes in the FEG were consistent and did not differ between the SSJIBL and BID groups. These changes suggest that the improvement in FEG is not entirely dependent on weight loss and reduced FI, indicating that other mechanisms may be involved. Numerous researchers believe that the improvement of diabetes after metabolic surgery is not only the result of weight loss, but also of the multiple mechanisms involved, such as BA and hormones (Madsbad et al., 2014; Pop et al., 2018). Wang et al. demonstrated that significant improvement in blood glucose levels after the jejunum-ileum circuit procedure was not related to weight loss (Wang et al., 2016). In addition, Liu et al. observed that changes in FI were inconsistent with improvements in blood glucose levels (Liu et al., 2019), which is consistent with our findings here.

We analyzed the relationship between BA and BW and observed that serum LCA and DCA were negatively correlated with BW. The serum LCA and DCA levels were higher in the SSJIBL group than in the SHAM group. These results suggest that high serum LCA and DCA levels are associated with decreased BW. Several studies have shown that adding non-12α-hydroxylated BAs (such as LCA) can effectively improve the obesity phenotype in mice (Wei et al., 2020; Li et al., 2022). Compared to the SSJIBL group, the serum LCA and DCA levels of rats in the BID group decreased more significantly, which may be one of the reasons why the weight loss effect in the BID group was not as great as that in the SSJIBL group. A previous study showed that weight gain in mice can be effectively prevented by administering UDCA and LCA (Wei et al., 2020). We also observed an increasing trend of LCA and DCA in the feces of the SSJIBL group. This further confirms that elevated serum BA levels are mainly increased through BA synthesis. Although several studies have suggested that BAs activate TGR5 to promote energy metabolism and thus reduce BW (Watanabe et al., 2006; Zietak and Kozak, 2016), the specific mechanism of BA and BW is still unclear. Furthermore, whether LCA and DCA regulate BW by means of energy metabolism or other mechanisms deserves further investigation.

We also analyzed the relationship between BA and AUC-OGTT and observed that LCA and DCA were negatively correlated with AUC-OGTT. In the current study, serum LCA and DCA levels were significantly elevated in the SSJIBL group than those in the SHAM group. In addition, we observed that the OGTT of the SSJIBL group was improved compared to that of the SHAM group at 14 days. These results suggest that SSJIBL improved glucose metabolism by increasing the serum levels of LCA and DCA. Elevated BA levels are closely associated with improved glucose levels (Kaska et al., 2016; Nemati et al., 2018), and Flynn et al. reported that simple BA diversion to the ileum has a favorable effect on improving glucose metabolism (Flynn et al., 2015). In the liver, the BA-FXR signaling pathway inhibits hepatic gluconeogenesis-related enzymes such as glucose-6-phosphatase and phosphoenolpyruvate carboxykinase, thereby regulating blood glucose levels (Zhang et al., 2006; Potthoff et al., 2011). In the intestine, BA activation of TGR5 promotes the secretion of GLP-1 by L cells, which stimulates insulin secretion by pancreatic β-cells, thus improving glucose metabolism (Katsuma et al., 2005; Thomas et al., 2009). Regrettably, the expression of gluconeogenesis-related enzymes and receptors was not detected in the current study. In addition, the lipid profiles were analyzed, and no significant reduction in total cholesterol was found in the SSJIBL and BID groups. Thus, elevated BA improves blood glucose metabolism, but not by lowering total cholesterol levels.

The present study had several limitations. First, this experiment was conducted for a short period of time, so a longer period will possibly help to better elucidate the mechanism. Second, we did not detect insulin levels and failed to assess the effect of BA on islet β-cell function. Finally, BA-related receptors were not measured in the present study. These problems warrant further systematic study.

In summary, elevated serum TBA levels may result from increased BA synthesis and are involved in glucose metabolism. LCA and DCA are closely associated with improved glucose metabolism; thus, they may serve as potential drugs and should be investigated in greater depth.

## Data Availability

The raw data supporting the conclusion of this article will be made available by the authors, without undue reservation.
